# Genomic and Genic Deletions of the FOX Gene Cluster on 16q24.1 and Inactivating Mutations of *FOXF1* Cause Alveolar Capillary Dysplasia and Other Malformations

**DOI:** 10.1016/j.ajhg.2009.05.005

**Published:** 2009-06-12

**Authors:** Paweł Stankiewicz, Partha Sen, Samarth S. Bhatt, Mekayla Storer, Zhilian Xia, Bassem A. Bejjani, Zhishuo Ou, Joanna Wiszniewska, Daniel J. Driscoll, Juan Bolivar, Mislen Bauer, Elaine H. Zackai, Donna McDonald-McGinn, Małgorzata M.J. Nowaczyk, Mitzi Murray, Tamim H. Shaikh, Vicki Martin, Matthew Tyreman, Ingrid Simonic, Lionel Willatt, Joan Paterson, Sarju Mehta, Diana Rajan, Tomas Fitzgerald, Susan Gribble, Elena Prigmore, Ankita Patel, Lisa G. Shaffer, Nigel P. Carter, Sau Wai Cheung, Claire Langston, Charles Shaw-Smith

**Affiliations:** 1 Dept of Molecular & Human Genetics, Baylor College of Medicine, Houston, TX 77030, USA; 2 Dept of Medical Genetics, Institute of Mother and Child, 01-211 Warsaw, Poland; 3 Dept of Pediatrics – Nutrition, Baylor College of Medicine, Houston, TX 77030, USA; 4 Institute of Child Health, WC1N 1EH London, UK; 5 Wellcome Trust Sanger Institute, Hinxton, CB10 1SA Cambridge, UK; 6 Signature Genomic Laboratories, LLC, Spokane, WA 99207, USA; 7 Division of Pediatric Genetics and Metabolism, University of Florida College of Medicine, Gainesville, FL 32610, USA; 8 Dept of Cardiology, Miami Children's Hospital, Miami, FL 33155, USA; 9 Dept of Genetics, Miami Children's Hospital, Miami, FL 33155, USA; 10 Division of Human Genetics, Children's Hospital of Philadelphia, PA 19104, USA; 11 Dept of Pathology and Molecular Medicine, McMaster University, Hamilton, Ontario L8S 3K9, Canada; 12 Dept of Medical Genetics, University of Washington, Seattle, WA 98195, USA; 13 Dept of Medical Genetics, Addenbrooke's Hospital, CB2 0QQ Cambridge, UK; 14 Dept of Pathology, Texas Children's Hospital, Baylor College of Medicine, Houston, TX 77030, USA

## Abstract

Alveolar capillary dysplasia with misalignment of pulmonary veins (ACD/MPV) is a rare, neonatally lethal developmental disorder of the lung with defining histologic abnormalities typically associated with multiple congenital anomalies (MCA). Using array CGH analysis, we have identified six overlapping microdeletions encompassing the FOX transcription factor gene cluster in chromosome 16q24.1q24.2 in patients with ACD/MPV and MCA. Subsequently, we have identified four different heterozygous mutations (frameshift, nonsense, and no-stop) in the candidate *FOXF1* gene in unrelated patients with sporadic ACD/MPV and MCA. Custom-designed, high-resolution microarray analysis of additional ACD/MPV samples revealed one microdeletion harboring *FOXF1* and two distinct microdeletions upstream of *FOXF1*, implicating a position effect. DNA sequence analysis revealed that in six of nine deletions, both breakpoints occurred in the portions of *Alu* elements showing eight to 43 base pairs of perfect microhomology, suggesting replication error Microhomology-Mediated Break-Induced Replication (MMBIR)/Fork Stalling and Template Switching (FoSTeS) as a mechanism of their formation. In contrast to the association of point mutations in *FOXF1* with bowel malrotation, microdeletions of *FOXF1* were associated with hypoplastic left heart syndrome and gastrointestinal atresias, probably due to haploinsufficiency for the neighboring *FOXC2* and *FOXL1* genes. These differences reveal the phenotypic consequences of gene alterations in *cis*.

## Introduction

Alveolar capillary dysplasia with misalignment of pulmonary veins (ACD/MPV) (Alveolar Capillary Dysplasia, Congenital; CACD [MIM 265380]) is a rare and lethal developmental disorder of the lung that affects both acinar structure and the intrinsic pulmonary vasculature, producing a constellation of histologic changes that when present together define the entity.^1^ Infants affected with ACD/MPV develop respiratory distress and severe pulmonary hypertension within the first two days of life, have no sustained response to supportive measures, and die of respiratory failure within the first month of life, although longer survivals and later presentations have been reported.^1^, ^2^, ^3^, ^4^, ^5^, ^6^, ^7^, ^8^, ^9^, ^10^ More than 80% of infants with ACD/MPV have additional malformations affecting the cardiac, gastrointestinal, and genitourinary systems. Intestinal malrotation is the most commonly observed of these anomalies, and hypoplastic left heart together with hypoplasia or coarctation of the aortic arch are the most common associated cardiovascular abnormalities.^10^, ^11^

Almost 200 ACD/MPV cases have been reported in the literature, approximately 10% having a familial association.^4^, ^5^, ^6^, ^10^, ^12^, ^13^, ^14^ The genetic etiology of ACD/MPV has remained elusive.^10^ Recently, *STRA6* (MIM 610745) has been implicated in a malformation syndrome that includes anophthalmia, additional malformations, and developmental lung abnormalities said to be ACD.^15^ However, the reported lung changes in children with *STRA6* mutations do not include misalignment of pulmonary veins, the defining histologic feature, and the clinical course is vastly different than that of ACD/MPV.

We initiated a study of the genetic basis of esophageal atresia and tracheo-esophageal fistula, ascertaining patients with malformations of this type, especially those that were associated with other congenital anomalies. We carried out an array comparative genomic hybridization (array CGH) screen to detect copy number variation (CNV) at critical genomic loci, particularly those harboring genes that were good candidates on the basis of their already established roles in foregut development in model organisms.

In the present study, we demonstrate a crucial role for *FOXF1* (MIM 601089) in human lung and intrinsic pulmonary vascular development by identifying inactivating mutations in patients with ACD/MPV. These patients have additional congenital malformations that together define a syndrome of ACD/MPV, intestinal malrotation, and urinary tract malformations. We show that patients with deletions harboring *FOXF1* and the neighboring *FOXC2* (MIM 602402) and *FOXL1* (MIM 603252) genes at 16q24.1 have not only ACD/MPV, as expected, but also distinct malformations comprising congenital heart defect, in particular hypoplastic left heart syndrome, and gastrointestinal atresias, including esophageal atresia, as well as urinary tract malformations and other malformations.

## Subjects and Methods

### Subject Recruitment

We obtained DNA samples from probands with ACD/MPV and their family members after obtaining their informed consent, using protocols approved by the Institutional Review Board for Human Subject Research at Baylor College of Medicine (H8712). The Cambridgeshire 4 Research Ethics Committee, UK, approved the study of a cohort of patients with esophageal atresia and associated malformations (reference 04/5/022). Patient D2 was ascertained via a study of prenatal malformations conducted at Addenbrooke's Hospital. This study was approved by Addenbrooke's Hospital Local Research Ethics Committee and by Cambridgshire 1 Research Ethics Committee, UK (reference 08/H0304/).

### Histopathology

Histologic slides of lung tissue obtained at autopsy (deletion cases D1, D3, D4, D8, and all four mutation cases M1–M4) or biopsy (deletion cases D9 and D10) were reviewed by C.L. for the diagnostic histologic features of ACD/MPV. All cases had slides stained with hematoxylin and eosin, and patient D1 also had elastic tissue stains.

### DNA Isolation

Patients' genomic DNA was extracted from peripheral blood via the Puregene DNA isolation kit (Gentra System, Minneapolis, MN, USA). DNA was extracted from frozen tissue and peripheral leukocytes via the Puregene DNA Extraction Kit, as well. Alternatively, DNA from paraffin blocks was isolated via the QIAGEN Kit in accordance with the vendor's instruction or as described previously.^10^

### Array CGH Analysis

Initial array CGH analysis was performed with the use of a 244K commercial array (Agilent Technologies, Santa Clara, CA, USA) in patient D1 and with an Affymetrix GeneChip 6.0 array in patient D2, in accordance with the manufacturers' instructions; no additional pathogenic CNVs were identified. Chromosomal microarray analysis was performed with the use of the V6.1 BAC-based array (patient D4), V6.3 OLIGO (patients D5 and D7), and V7.2 OLIGO (patient D3), designed by Baylor Medical Genetics laboratories and manufactured by Agilent Technology as previously described.^16^, ^17^ Array CGH in patient D5 was performed with the use of the BAC clone SignatureChipWG whole-genome microarray, in patient D6 with the use of the SignatureChipOS, a 105K-feature whole-genome microarray (made for Signature Genomic Laboratories by Agilent Technologies), in accordance with the manufacturer's instructions. Patient D5 was also analyzed with the use of the Affymetrix 250K SNP array.

Whole-genome high-resolution oligonucleotide microarray CGH analyses in patients D1–D9, for fine mapping of the sizes and extents of the deletions, and in subject D10, for confirmation of the deletion, were performed with the NimbleGen array HG18_WG_CGH_v1 with 385,000 or 2.1M oligonucleotides (NimbleGen Systems, Madison, WI, USA), in accordance with the manufacturer's instructions.

Custom 16q24 region-specific high-resolution 44K microarrays were designed with the use of eArray (Agilent Technologies) and used for CNV screening in 14 mutation-negative patients with ACD/MPV.

### Fluorescence In Situ Hybridization Analysis

Confirmatory fluorescence in situ hybridization (FISH) analysis in patients D2–D5 was performed via standard procedures.

### Long-Range Polymerase Chain Reaction and DNA Sequencing

Long-range polymerase chain reaction (LR-PCR), performed in accordance with the manufacturer's instructions (Takara Bio, Japan), amplified the predicted junction fragments from the breakpoint regions in patients D1–D9. PCR products were purified with the PCR Purification Kit (QIAGEN, Valencia, CA, USA) and bidirectionally sequenced by Sanger di-deoxynucleotide sequencing (Lone Star Labs, Houston, TX, USA).

### Mutation Analysis of the *FOXF1* Gene

Overlapping amplicons covering the entire coding region of exons 1 and 2 of *FOXF1* were amplified and sequenced by conventional Sanger di-deoxynucleotide sequencing (Lone Star Labs, Houston, TX; Molecular Core Lab, BCM, Houston, TX). DNA sequences were analyzed by comparison with reference sequence (NM_001451.2) with the use of Sequencher v4.2 (GeneCodes, Ann Arbor, MI, USA). None of the identified mutations was found in 150 ethnically matched control chromosomes. Individual primer sequences and PCR conditions are available on request.

### Mutation Cloning

A 1482 bp region covering the mutation and an informative heterozygous (C/G) SNP, rs2078304, in patient M4 was amplified by LR-PCR and cloned with the Copy Control Cloning Kit (Epicenter Biotechnologies, Madison, WI, USA). Clone DNA was isolated with the Qiprep Miniprep Kit (QIAGEN, Valencia, CA, USA) and sequenced by conventional Sanger di-deoxynucleotide sequencing (Lone Star Labs, Houston, TX, USA).

### Polymorphic SNP and Microsatellite Marker Analysis

SNP markers rs12596341, rs10660430, rs11398689, rs1364225, rs1424019, rs9941308, rs58016760, rs1364224, rs1424016, rs58557724, and rs1064259 and the microsatellite marker between chr16:84,723,490–84,723,542 were amplified for parental studies via routine methods with PCR primers obtained from Sigma (Woodlands, TX, USA). The SNP PCR products were bidirectionally sequenced (Lone Star Labs, Houston, TX, USA). The microsatellite amplicons were separated on 8% polyacrylamide gels and visualized by ethidium bromide staining. The loci order, locations, and sequences were obtained from the UCSC Genome Browser database.

### Bioinformatics and In Silico Sequence Analysis

Genomic sequence based on the oligonucleotide coordinates from the array CGH experiment was downloaded from the UCSC genome browser (build 36, March 2006) and assembled with the Sequencher v4.2 software. Interspersed repeat sequences were analyzed by RepeatMasker.

## Results

### Initial Array CGH Screening

**Figure 1:**
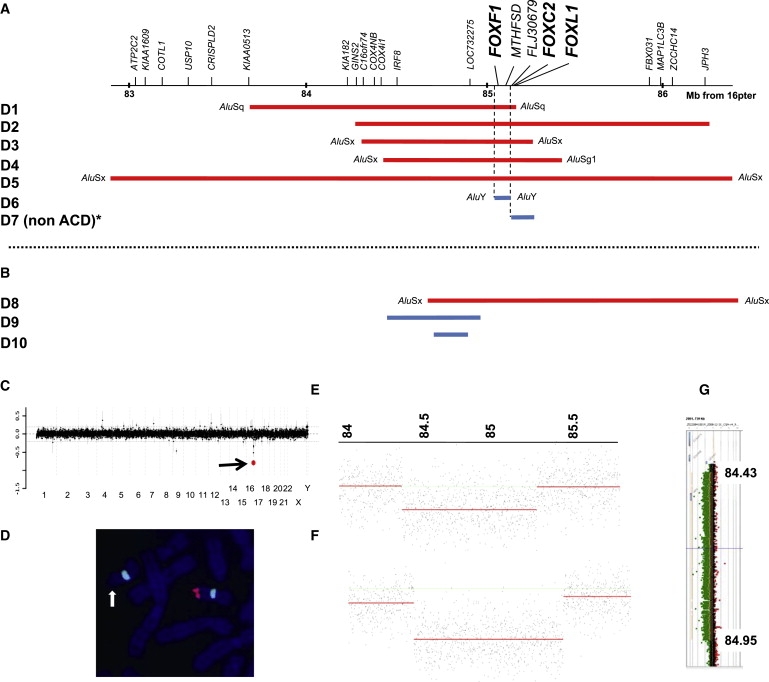
Summary of the Results in Patients with Microdeletions in 16q24.1q24.2 (A) Schematic representation of the genomic region harboring the FOX transcription gene cluster, *FOXF1*, *FOXC2*, and *FOXL1*, showing the extent and gene content of the regions deleted in seven patients analyzed by array CGH (D1–D7). A non-ACD/MPV patient, D7, with a deletion of *FOXC2* and *FOXL1* and an intact *FOXF1*, is also shown. (B) Microdeletions identified via a custom designed 16q24-specific high-resolution 4×44K Agilent array in three patients with ACD/MPV (D8–D10). Note that the microdeletion in subject D8 contains *FOXF1*, whereas those in patients D9 and D10 are located upstream of *FOXF1*, indicating a position effect on this gene. Group 1 patients are indicated in red, group 2 in blue (see main text for explanation). In six out of nine deletions analyzed, breakpoints map within *Alu* repetitive elements, suggesting MMBIR/FoSTeS or NAHR mechanisms of formation. (C) Targeted array CGH plot obtained with V7.2 OLIGO (Agilent 105K) in patient D3, showing a deletion in 16q24.1 (red mark designated by arrow). (D) Metaphase chromosomes of patient D4 after FISH with a 16q24-specific BAC clone, RP11-542M13 (red), and a control chromosome 16 alfa satellite (Vysis) probe (green), showing absence of signal (arrow) on one chromosome 16, consistent with a heterozygous deletion. (E and F) NimbleGen whole-genome oligonucleotide array CGH profiles for subjects D3 (E) and D4 (F). (G) The ∼524 kb deletion in patient D9, located ∼52 kb upstream of *FOXF1*, was detected via a custom designed 16q24-specific high-resolution 4×44K Agilent array. Green dots shifted to the left between 84.43–84.95 represent the deleted segment.

Using BAC clone and oligonucleotide array CGH, we identified overlapping microdeletions in 16q24.1q24.2, ranging in size from ∼100 kb to ∼3.5 Mb, in seven patients referred for high-resolution genome analysis (Figures 1A and 1C, Table 1). The first of these patients (D1) was ascertained through our study of the genetic basis of esophageal atresia, for which, to date, 80 syndromic cases have been studied. The remainder were ascertained by queries of databases of patients referred for high-resolution genome analysis for a variety of reasons. Parental samples were available in six out of seven patients (not D2). One deletion (D7) was inherited from a phenotypically abnormal parent; the remaining five were de novo, supporting their likely pathogenic effect. No deletion CNVs in this genomic region were found, either in the Database of Genomic Variants or in the BCM and Signature Genomics databases of over 30,000 patients studied by array CGH. Five of the seven deletions (D2–D5 and D7) were independently verified by FISH analysis (Figure 1D). High-resolution oligonucleotide array CGH (385K or 2.1M NimbleGen) confirmed the deletions and enabled breakpoint characterization in all seven cases (Figures 1E and 1F).

**Table 1:** Summary of the Clinical Findings in Patients with FOX Gene Cluster Microdeletions in 16q24.1q24.2

Patient	Deletion	M/F	Gest. (wks)	LS	Birth Wt. (g)	Respiratory Findings	Cardiac Findings	Gastrointestinal Findings	Genitourinary Findings	Vertebral/Axial Findings	Other Findings
D1	∼1.5 Mb *FOXF1, FOXC2, FOXL1*	F	28	1 day	1091	ACD/MPV, pulmonary lymphangiectasia	PDA	Esophageal atresia, tracheo-esophageal fistula; abnormal placement of anus	-	-	Single umbilical artery
D2	∼2.0 Mb *FOXF1, FOXC2, FOXL1*	M	22; TOP	N/A	NK	-	HLHS	-	Dilated renal pelvices	-	-
D3	∼0.9 Mb *FOXF1, FOXC2, FOXL1*	M	38; elective C-section	40 days	2900	ACD/MPV; persistent pulmonary hypertension; ECMO dependent (veno-veno)	Tetralogy of Fallot; PDA	Duodenal atresia with annular pancreas; imperforate anus	Bilateral hydronephrosis, mod right, severe left	-	Single umbilical artery
D4	∼1.0 Mb *FOXF1, FOXC2, FOXL1*	F	37; elective C-section	15 days	-	ACD/MPV; left pneumothorax; hypoxemia; Surfactant therapy; ECMO	HLHS	-	Mild uretero-pelvic caliectasis	T11 butterfly vertebra	Cleft lip, cleft palate, brachycephaly; single umbilical artery
D5	∼3.5 Mb *FOXF1, FOXC2, FOXL1*	M	-	18 days	-	ECMO dependent	IAA, dilated PA, large PDA, decreased size left ventricle, pulmonary hypertension	-	Bilateral renal pelviectasis	Posterior rib fusions: 10/11 (right side); 9/10 and 11/12 (left side)	-
D6	∼103 Kb *FOXF1, MTHFSD*	M	35	5 days	1700	Respiratory distress requiring inhaled nitrous oxide, hydrocortisone, prostaglandin and FiO2 100%, attributed to RDS	AVSD, ostium primum ASD, small VSD, PDA, mildly hypoplastic LV, bicuspid AV,	Possible duodenal atresia, possible intestinal malrotation	Moderate bilateral pelvicaliectasis, mild to moderate proximal urethral dilatation; probable mild to moderate distal ureteral dilatation	-	Absent spleen; transverse orientation of the liver, compatible with abdominal heterotaxy
D7	131 Kb, *FOXC2* and *FOXL1*	F	-	Alive at 3 yrs	-	-	Spontaneous ASD closing	Diastasis recti (6 cm at 14 months of age)	Dilated and tortuous ureters bilaterally with left hydronephrosis	-	CNS, ventriculomegaly, Chiari malformation and syrinx, meningeal cysts at T2-T3, developmental delay, o/e hypertelorism and broad forehead similar to mother, low-set ears, CAL R buttock and L lower scapula, healing capillary hemangioma L buttock
D8^a^	∼1.8 Mb *FOXF1, FOXC2, FOXL1*	F	term	25 days	3676	ACD/MPV; persistent pulmonary hypertension; lung biopsy done	PDA, ligated; structurally normal heart by echocardiogram	Adhesions between bowel loops, second part of duodenum and gallbladder; no malrotation seen at laparotomy	-	-	-
D9	524 Kb, Upstream of *FOXF1*	F	term	20 days	3742	ACD/MPV; persistent pulmonary hypertension, 2 courses of ECMO days 2-7 and again beginning on day 10. Lung biopsy at 17 days	Multiple normal echocardiograms	Suspected intestinal malrotation; imperforate anus	Bicornuate uterus with cervical duplication	Multiple butterfly vertebrae	-
D10	145 Kb, Upstream of *FOXF1*	F	term	13 days	3600	ACD/MPV respiratory distress with pulmonary hypertension, requiring mechanical ventilation, HFOV, inhaled nitric oxide, ECMO. Lung biopsy at 12 days	Echocardiogram on day 2 showed changes related to severe pulmonary hypertension and small PDA with no other abnormality	-	-	-	-

Abbreviations are as follows: M, male; F, female; Gest., gestation; LS, lifespan; TOP, elective termination of pregnancy; PA, pulmonary artery; ASD, atrial septal defect; VSD, ventricular septal defect; AVSD atrioventricular septal defect; LV, left ventricle; AV, aortic valve; IAA, interrupted aortic arch; HLHS, hypoplastic left heart syndrome; ECMO, extracorporeal membrane oxygenation; PDA, patent ductus arteriosus; RDS, respiratory distress syndrome; HFOV, high frequency oscillatory ventilation; CAL, cafe-au-lait patch.

a This patient was described in Sen et al.^10^

The deletions centered around the FOX transcription factor gene cluster at 16q24.1. All but one harbored *FOXF1*, a gene with a role in lung and foregut development ascertained on the basis of previous studies of mice.^18^, ^19^, ^20^ Patient D7 had an ∼131 kb deletion encompassing *FOXC2* and *FOXL1* but not *FOXF1*.

### Clinical Characterization

**Figure 2:**
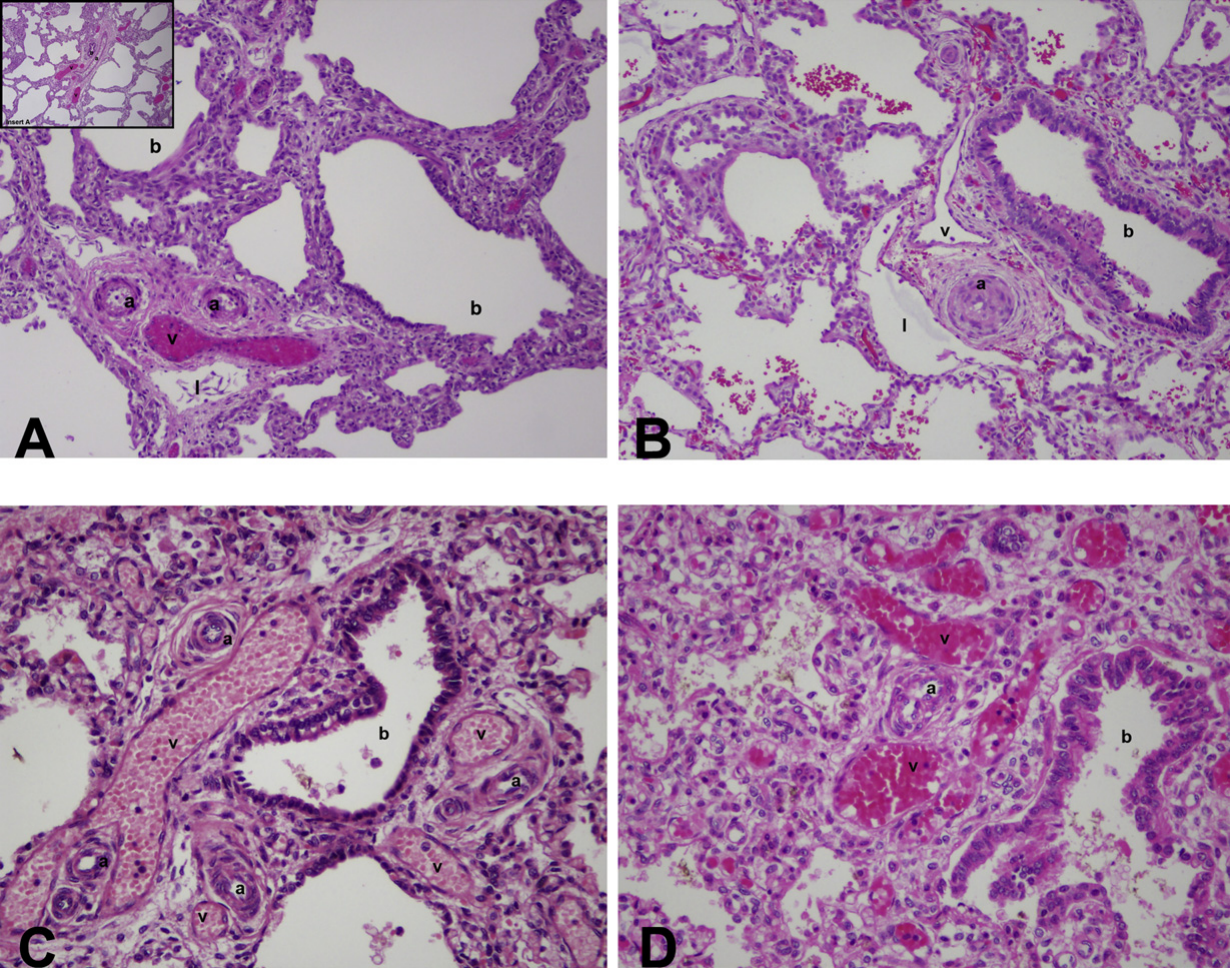
Histopathology Studies Histopathology studies show that all deletion and mutation patients have characteristic changes of ACD/MPV, with medial hyperplasia of small pulmonary arteries, abnormally positioned pulmonary veins adjacent to membranous and terminal bronchioles and coursing with small pulmonary arteries (misalignment), lobular underdevelopment, and deficient numbers of normally positioned airspace wall capillaries with abnormal enlarged and centrally placed thin-walled vascular channels in airspace walls. The deletion cases often show marked airspace enlargement, thinner airspace walls, and pulmonary lymphangiectasis; these findings are uncommon in mutation cases. Hematoxylin and eosin, initial magnification 25x. (A) Patient D3 with *FOXF1* deletion. Note two small pulmonary artery branches with moderately thickened smooth muscle (a), an adjacent malpositioned congested pulmonary vein (v), and a dilated lymphatic channel (l) all located adjacent to a dilated terminal bronchiole (b). In the inset, this same abnormal vascular configuration is seen adjacent to a membranous bronchiole and the lobular parenchyma is formed of markedly enlarged and simplified airspaces with thinner walls, compared to mutation cases. (B) Patient D9 with deletion upstream of *FOXF1*. Small pulmonary arteries (a) have strikingly thickened medial smooth muscle and only pin-point lumens; they share a common connective tissue sheath with pulmonary vein branches (v) that are neither dilated nor congested and both are adjacent to a membranous bronchiole (b). Normally positioned capillaries are not seen in airspace walls, although there are more centrally placed congested thin-walled vascular channels. A prominent lymphatic (l) is adjacent to the artery/vein combination. Airspaces appear prominently enlarged. (C) Patient M2 with frameshift mutation in exon 1. Multiple thick-walled small pulmonary arteries (a) and dilated congested malpositioned pulmonary veins (v) often share the same connective tissue sheath adjacent to a small bronchiole. The lobular parenchyma is formed of enlarged, simplified and poorly subdivided airspaces; however airspace enlargement is less dramatic than in the deletion cases. (D) Patient M3 with frameshift mutation in exon 2. Thick-walled small pulmonary arteries (a) and adjacent dilated and congested veins (v) are located next to a terminal bronchiole (b). The lobular parenchyma is underdeveloped with simplified and poorly subdivided airspaces separated by thickened septa in which there are dilated and congested central vascular channels, but only rare capillaries.

Of the six patients with chromosomal deletions harboring *FOXF1* (D1–D6), five (D1 and D3–D6) died from pulmonary insufficiency in the first two months of life, and the mother of a sixth (D2) underwent elective termination of pregnancy at 22 weeks. Three patients (D1, D3, and D4) had ACD/MPV documented by histopathological examination (Figure 2A). Two other patients (D5 and D6) had severe respiratory distress, raising the possibility of ACD/MPV, but no postmortem examination was performed. Cardiac malformations were present in five of the six patients; two patients (D2 and D4) had a hypoplastic left heart, and a third (D5) had a small left ventricle. One patient (D1) manifested esophageal atresia with tracheo-esophageal fistula, whereas duodenal and anal atresias were present in another patient (D3). Five patients (D2–D6) had abnormalities consistent with urinary tract obstructive lesions, ranging from uretero-pelvocaliectasis to severe hydronephrosis. The phenotype in patient D7 was clearly distinct from that in the other six patients. This patient did not have respiratory insufficiency in the neonatal period and was still alive at the age of 3 years, having presented with atrial septal defect, bilaterally dilated and tortuous ureters with left hydronephrosis, diastasis recti, and developmental delay (Figure 2A and Table 1). The deletion was inherited from the patient's mother, whose sole recognized phenotypic abnormality was the presence of an umbilical hernia.

### Mutation Analysis of the *FOXF1* Gene

**Figure 3:**
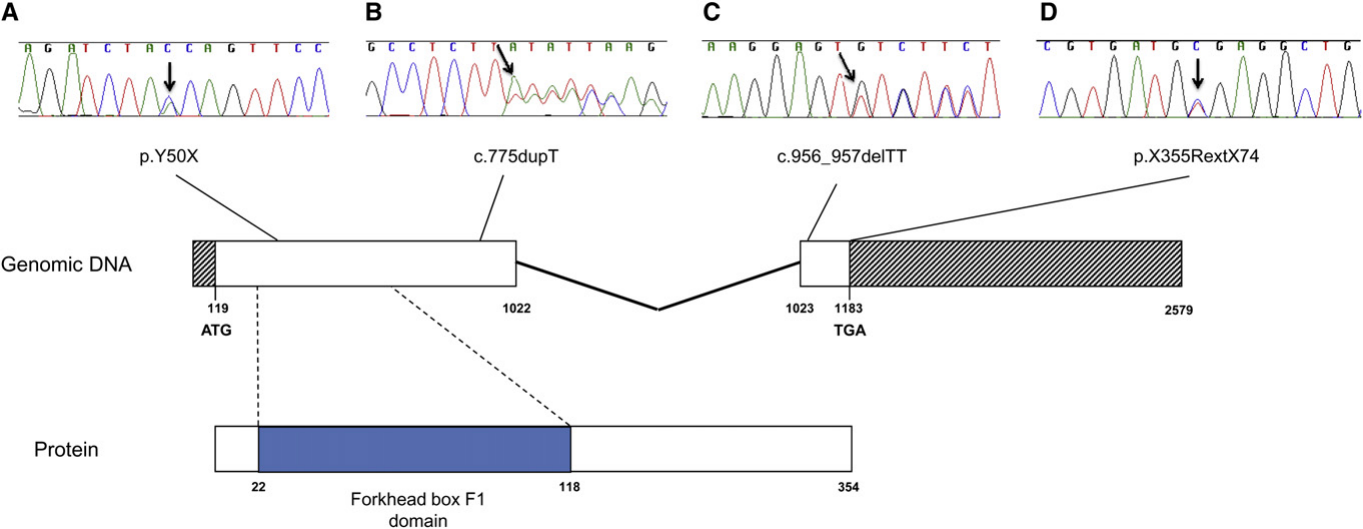
Genomic and Protein Structure of FOXF1 and DNA Sequencing Chromatograms Genomic and protein structure of FOXF1 and DNA sequencing chromatograms showing four mutations in the coding sequence of *FOXF1* identified in patients with ACD/MPV. (A) A nonsense mutation c.150C > A; p.Y50X in exon 1 in patient M1. (B) A frameshift mutation c.775 dupT; p.Y259Lfs11X in exon 1 in subject M2. (C) A frameshift mutation c.956_957 delTT; p.F319CfsX66 in exon 2 in patient M3. (D) A no-stop mutation c.1063T > C; p.X355RextX74 in exon 2 in individual M4. The base numbering for the *FOXF1* gene refers to A of the second ATG start codon as position 1 (nucleotide 119 of GenBank accession number NM_001451.2).^34^

Two lines of evidence suggested that *FOXF1* haploinsufficiency might be responsible for ACD/MPV in our initial cohort. First, patients with deletions harboring *FOXF1* (D1–D6) had a severe respiratory phenotype (confirmed histologically as ACD/MPV in three cases), whereas a patient with a deletion harboring *FOXC2* and *FOXL1* but not *FOXF1*, patient D7, did not. Second, mice haploinsufficient for *Foxf1* have abnormal alveolar development, notwithstanding the fact that the histological changes are not identical to those of ACD/MPV.^18^, ^19^ We therefore sequenced *FOXF1* in a cohort of 18 patients with ACD/MPV and other malformations.^10^ We identified four de novo heterozygous mutations in the coding sequence of *FOXF1* in four unrelated patients with sporadic ACD/MPV. Patient M1 had a nonsense mutation (c.150C→A; p.Y50X) in exon 1; patient M2 had a frameshift mutation (c.775dupT; p.Y259Lfs11X) in exon 1; patient M3 had a frameshift mutation (c.956_957delTT; p.F319CfsX66) in exon 2 adding 29 amino acids to the protein, as predicted by conceptual translation; and patient M4 had a T→C substitution in the first base of the stop codon, a no-stop mutation (c.1063T→C; p.X355RextX74) adding 73 amino acids to the protein, as predicted by conceptual translation (Figures 3A–3D). All four patients had associated malformations, including a partial atrioventricular canal defect (1/4 cases), patent ductus arteriosus (1/4), bowel malrotation (3/4), a congenital short bowel (1/4), an annular pancreas (1/4), and urinary tract malformations (3/4) (Table 2).

**Table 2:** Summary of the clinical findings in patients with *FOXF1* mutations

Patient	Mutation	M/F	Gest. (wks)	LS	Birth Wt. (g)	Respiratory Findings	Cardiac Findings	Gastrointestinal Findings	Genitourinary Findings	Vertebral/Axial Findings	Other Findings
M1^a^	Exon 1, Nonsense	M	32	10 days	2470	ACD/MPV; bilobed right lung	Partial atrio-ventricular canal defect, PDA	Intestinal malrotation; Annular pancreas and duodenal stenosis	Bilateral hydronephrosis, hydroureter, dilatation urinary bladder	-	-
M2	Exon 1, Frameshift	M	32	12 hrs	2460	ACD/MPV	-	-	Obstructive renal dysplasia	-	-
M3	Exon 2, Frameshift	F	35	1 day	2490	ACD/MPV; partial anomalous pulmonary venous connection with absent left upper lobe pulmonary vein	-	Malrotation bowel; congenital short bowel; small omphalocele	Bilateral severe hydronephrosis, hydroureter, and bladder dilatation	-	Micrognathia, low set ears
M4	Exon, 2, No-stop	M	38	47 days	5057	ACD/MPV	-	Intestinal malrotation, Meckel's diverticulum	-	-	-

Abbreviations are as follows: M, male; F, female; Gest., gestation; LS, lifespan; PDA, patent ductus arteriosus.

a This patient was described in Sen et al.^10^

### Custom-Designed Array CGH

We hypothesized that some of the cases of ACD/MPV in the cohort might be due to whole-gene deletions, as was seen for patients D1–D6. We therefore designed a 16q24 region-specific high-resolution 44K oligonucleotide microarray and used it to screen the 14 mutation-negative patients from our ACD cohort. This analysis revealed an ∼1.8 Mb microdeletion harboring *FOXF1* in patient D8. In addition, two microdeletions, one ∼524 kb (patient D9) and one ∼145 kb (patient D10) in size, located ∼52 kb and ∼259 kb upstream of *FOXF1*, respectively, were detected (Figures 1B and 1G, Table 1). All three rearrangements arose de novo.

### Histopathology

Of 14 patients, ten had lung tissue available for histologic review. All ten showed the constellation of changes required for the histologic diagnosis of ACD/MPV (Figure 2). Many also showed other superimposed changes; consequences of respiratory failure and therapeutic support, terminal events, postmortem changes, and focal inflammation (rarely). However, these did not obscure the diagnostic hallmarks of ACD/MPV. Pulmonary lymphangiectasis was evident in all analyzed deletion cases (D1, D3, D4, and D8–D10), as well as in one (M2) of four examined patients with mutations. In the patients with lung biopsy but without autopsy (D9 and D10), information related to other malformations was based on clinical and/or imaging observations without pathologic confirmation; for those with neither lung biopsy nor autopsy (D2, D5, and D6), lung histology remains unknown and information related to other malformations is based on clinical and/or imaging observations only. No pulmonary tissue is available from patient D7, and that patient remains alive at three years of age, without respiratory symptomatology.

### DNA Sequence Analysis

DNA sequence analysis enabled breakpoint sequencing in patients D1–D9. This confirmed the deletions and revealed that in patients D1, D3–D6, and D8, both breakpoints occurred in the portions of *Alu* elements showing eight to 43 base pairs (bp) of perfect microhomology. In patients D2 and D7, microhomologies of 4 and 3 bp, respectively, were found, and in patient D9, a 3 bp AGC insertion was identified at the junction fragments (Table 3).

**Table 3:** Array CGH-Detected Microdeletions in 16q24

Patient	Initial Array CGH Detection	FISH Verification	Parental Origin of Deletion	High-Resolution Array CGH Verification and Characterization	Coordinates	Size	Micro-homology (bp)	Mechanism of Formation
D1	244 K (Agilent Technologies)	ND	de novo maternal	385 K (NimbleGen)	83,705,765–85,204,004	1,498,239 bp	17	*Alu-Alu* MMBIR/ FoSTeSx1 or NAHR
D2	Affymetrix SNP 6.0	RP11-565I1	ND	385 K (NimbleGen)	84,275,154–86,275,754	2,000,600 bp	4	NHEJ or MMBIR/FoSTeSx1
D3	CMA V7.2 OLIGO	RP11-542M13	de novo maternal	2.1 M (NimbleGen)	84,374,208–85,277,007	902,799 bp	18	*Alu-Alu* MMBIR/FoSTeSx1 or NAHR
D4	CMA V6.1 BAC	RP11-542M13	de novo maternal	2.1 M (NimbleGen)	84,402,571–85,435,712	1,033,141 bp	8	*Alu-Alu* MMBIR/FoSTeSx1 or NAHR
D5	SignatureChipWG	RP11-463O9	de novo ND	385 K (NimbleGen) Affymetrix 250K	82,908,199–86,405,076	3,496,877 bp	12	*Alu-Alu* MMBIR/FoSTeSx1 or NAHR
D6	SignatureChipOS	ND	de novo ND	2.1 M (NimbleGen)	85,051,032–85,153,914	102,882 bp	19	*Alu-Alu* MMBIR/FoSTeSx1 or NAHR
D7	CMA V6.3 OLIGO	RP11-58A18	inherited maternal	2.1 M (NimbleGen)	85,157,843–85,288,903	131,060 bp	3	NHEJ or MMBIR/FoSTeSx1
D8	4×44 K (Agilent Technologies)	ND	de novo maternal	385 K (NimbleGen)	84,648,160–86,478,255	1,830,095 bp	43	*Alu-Alu* MMBIR/FoSTeSx1 or NAHR
D9	4×44 K (Agilent Technologies)	ND	de novo maternal	385 K (NimbleGen)	84,425,269–84,949,662	524,393 bp	1	NHEJ AGC insertion
D10	4×44 K (Agilent Technologies)	ND	de novo maternal	385 K (NimbleGen)	∼ 84,698–84,843 kb	∼ 145 kb	ND	ND

Nucleotide numbering is based on human genome, UCSC Genome Browser, build 36 (March 2006). ND indicates not determined.

### Parental Origin of Deletions and Mutation

We investigated, when possible, the parent of origin, and we showed by microsatellite polymorphism and SNP studies that the deletions in patients D1, D3, D4, D8, D9, and D10 are maternal in origin. In patient M4, DNA sequencing of the cloned PCR product revealed that the no-stop mutation c.1063T→C was associated with the C allele inherited from the patient's father, whereas the G allele was associated with the wild allele, demonstrating that the mutation occurred on the paternal chromosome (Table S1).

## Discussion

Our report of deletions and point mutations within the FOX transcription factor gene cluster at 16q24.1 highlights the differing phenotypic consequences of gene alterations in *cis*. The patients described in this report can be classified into three groups on the basis of genotype and phenotype. Patients in group 1 have deletions that encompass all three FOX genes in the 16q24.1 cluster—*FOXF1*, *FOXC2*, and *FOXL1*—as well as *MTHFSD* (genomic deletions). Group 2 patients have deletions spanning either none (patients D9 and D10) or some (patients D6 and D7; genic deletions) but not all of the genes in the FOX cluster. Group 3 patients comprise those with inactivating point mutations in *FOXF1*. All group 1 and group 3 patients with histologic evaluation had ACD/MPV, but there were consistent differences in the spectrum of associated malformations between the two groups. All six group 1 patients had cardiovascular malformations. These ranged in severity from patent ductus arteriosus (PDA) (D1, D3, D5, and D8) through interrupted aortic arch (D5) and tetralogy of Fallot (D3) to reduced left ventricular size (D5) and hypoplastic left heart (D2 and D4). Just one of the four patients in group 3 (M1) had a cardiac malformation, a partial AV canal defect, together with PDA. Group 1 patients had gastrointestinal atresias (esophageal in D1, duodenal and anal in D3), whereas group 3 patients had intestinal malrotation or congenital short bowel. As expected, patients in group 2 were less readily classifiable. Patients with deletions, either upstream of (D9 and D10) or encompassing (D6) *FOXF1*, had ACD/MPV; one patient (D7) with a downstream deletion encompassing just *FOXC2* and *FOXL1* did not. Likewise, malformations occurring in this group were of group 1 type, such as hypoplastic left heart and imperforate anus, and group 3 type, such as intestinal malrotation. Additional malformations not seen in either group 1 or group 3 occurred in this group: possible abdominal heterotaxy in patient D6, Chiari malformation and meningeal cysts in patient D7.

We propose that ACD/MPV results from haploinsufficiency of *FOXF1* and that the differences in the associated malformations between groups 1 and 3 relate to haploinsufficiency for *FOXC2* and *FOXL1*. Point mutations in *FOXC2*, probably leading to its haploinsufficiency, are responsible for lymphoedema-distichiasis syndrome;^21^, ^22^ a small proportion of these patients also have congenital heart defects (6.8% in one series,^21^ including tetralogy of Fallot and ventricular septal defect but not hypoplastic left heart). Mice with targeted inactivation of the orthologous *Foxc2* have interrupted aortic arch, as well as defects of the skull and vertebral column.^23^, ^24^ Haploinsufficiency of *FOXL1* in humans has not previously been described. Targeted disruption of *Foxl1* in mice is associated with aberrant epithelial cell positioning in the small intestine, hyperplasia of the gastric mucosa and retarded growth.^25^, ^26^, ^27^ *MTHFSD* is a methylene tetrahydrofolate synthase domain-containing gene of unknown function.

The lack of intestinal atresia in group 3 suggests that either *FOXC2* or *FOXL1* may also contribute to this phenotype in humans, despite the fact that, in mice, haploinsufficiency for *Foxf1* alone is associated with esophageal atresia. It will be fascinating to determine whether deletions or inactivating mutations contribute to malformations reported here when they occur in isolation, in particular for hypoplastic left heart syndrome and intestinal malrotation, for which no gene has yet been causally implicated. The presence of overlapping deletions upstream of the FOX cluster in two patients (D9 and D10) with ACD/MPV strongly suggests that critical regulatory sequences are harbored within the minimal interval, and future work will focus on the identification of any evolutionarily conserved elements within this interval and the elucidation of their functional role.

Although the abnormal lung phenotype of *Foxf1*^+/−^ mice has some overlap with that of ACD/MPV in humans with alveolarization abnormalities, paucity of alveolar capillaries and pulmonary lymphangiectasia being common to both, there is no evidence of MPV, the defining histologic feature of ACD/MPV, in the mutant mice. Two different mouse models showed that *Foxf1*^−/−^ embryos die by 8.5 days postcoitum (dpc) from defects in extraembryonic mesoderm development.^18^, ^19^, ^28^ Interestingly, in one model, half of the newborn *Foxf1*^+/−^ mice had reduced (20% of wild-type) levels of pulmonary Foxf1, died from pulmonary hemorrhage, and showed severe defects in alveolarization and vasculogenesis (lung immaturity, hypoplasia) with fusion of the right lung lobes and lobar arteries, as well as an abnormal gall bladder, narrowing of the esophagus and trachea, esophageal atresia with tracheo-esophageal fistula, and defects in liver and lung repair. The other half of the *Foxf1*^+/−^ mice had normal Foxf1 levels and normal lung morphology, interpreted as a “compensation” for the alveolarization defect.^28^ We suggest that the occurrence of these two distinct populations of mice is consistent with imprinting of *Foxf1*, in which the expression level of *Foxf1* in *Foxf1*^+/−^ mice is dependent upon the parent of origin of the normal *Foxf1* allele. Supporting this notion, the expression from the *Foxf1* promoter has been shown to be regulated by tissue-specific methylation^29^ and *FOXF1* was computationally predicted to be paternally imprinted in humans.^30^ However, the facts that all six de novo deletions that we studied for the parental origin arose on a maternal chromosome and one mutation of *FOXF1* (in patient M4) occurred on a paternal chromosome, as well as the observation of a relatively normal phenotype associated with paternal uniparental disomy 16,^31^ argue against *FOXF1* imprinting in humans; nevertheless, a complex tissue-specific genomic imprinting cannot be excluded.

*Foxf1* has been shown to be downregulated in Shh^−/−^ mice, probably through Gli proteins,^32^ and activated by bone morphogenetic protein 4 (BMP4).^32^, ^33^ In *Foxf1*^−/+^ mice, a diminished expression of *Vegfa*, *Vegfr2*, *Bmp-4*, *Tbx*, *Lklf*, *Fgf-10*, *Gli3*, and *Notch-2* has been found.^19^, ^28^, ^34^, ^35^, ^36^ Some of these genes, critical for lung development, may be mutated in patients with ACD/MPV without detectable abnormalities in *FOXF1*.

DNA sequence analysis of the deletion breakpoints in nine patients (D1–D9) revealed that in six patients (D1, D3–D6, and D8), both breakpoints map in the portions of *Alu* elements showing perfect microhomology (eight to 43 base pairs) (Table 3), suggesting replication error MMBIR/FoSTeS as a mechanism of their formation.^37^ Alternatively, these deletions might have occurred via nonallelic homologous recombination (NAHR). In patients D2, D7, and D9, the presence of short microhomologies (D2 and D7) and AGC insertion (D9) at the junction fragments indicate that these deletions probably arose via nonhomologous end joining or the MMBIR/FoSTeS mechanism.^37^

Our data may contribute to a better understanding of the pathogenesis of malformations seen in patients with the VACTERL (vertebral, anal, cardiac, tracheo-esophageal, renal and limb malformations) association (MIM 192350).^38^ Cardiac malformations, esophageal and anal atresia, renal and vertebral/axial malformations, and a single umbilical artery appear to be common to both VACTERL association and 16q24.1 microdeletion. However, although lung malformations have been reported occasionally in cases of VACTERL association, ACD/MPV has not been described in this context. We suggest that microdeletions at 16q24.1 should be considered in cases of apparent VACTERL association for which there is a severe respiratory distress and/or neonatal lethality. We propose that patients with intestinal atresia, isolated diastasis recti, or umbilical hernia should be screened for mutations and deletions in *FOXL1*. Given that *FOXF1* maps only ∼3.7 Mb from 16qter, we also suggest that a balanced rearrangement involving 16qter (e.g., submicroscopic subtelomeric reciprocal translocation) should be excluded in parents with an ACD/MPV-affected child from whom no material is available for testing.

## Web Resources

The URLs for data presented herein are as follows:

Database of Genomic Variants, http://projects.tcag.ca/variation/
Online Mendelian Inheritance in Man (OMIM), http://www.ncbi.nlm.nih.gov/Omim/
RepeatMasker, http://www.repeatmasker.org
University of California, Santa Clara (UCSC) Genome Browser, http://genome.ucsc.edu/

## Supplemental Data

Document S1. Two Tables
