# Supplementary material for: Genomic and Genic Deletions of the FOX Gene Cluster on 16q24.1 and Inactivating Mutations of FOXF1 Cause Alveolar Capillary Dysplasia and Other Malformations
Source: Am J Hum Genet. 2009 Jun 12;84(6):780–91. doi: 10.1016/j.ajhg.2009.05.005 (PMC2694971; doi:10.1016/j.ajhg.2009.05.005)
Supplement: Document S1. Two Tables [file mmc1.pdf]

AJHG, Volume *84*

Supplemental Data

Genomic and Genic Deletions of the FOX Gene Cluster on 16q24.1 and Inactivating Mutations of FOXF1 Cause Alveolar Capillary Dysplasia and Other Malformations

Paweł Stankiewicz Partha Sen, Samarth S. Bhatt, Mekayla Storer, Zhilian Xia, Bassem A. Bejjani, Zhishuo Ou, Joanna Wiszniewska, Daniel J. Driscoll, Juan Bolivar, Mislen Bauer, Elaine H. Zackai, Donna McDonald-McGinn, Małgorzata M.J. Nowaczyk, Mitzi Murray, Tamim H. Shaikh, Vicki Martin, Matthew Tyreman Ingrid Simonic, Lionel Willatt, Joan Paterson, Sarju Mehta, Diana Rajan, Tomas Fitzgerald, Susan Gribble, Elena Prigmore, Ankita Patel, Lisa G. Shaffer, Nigel P. Carter, Sau Wai Cheung, Claire Langston, and Charles Shaw-Smith

Table S1. Summary of the Results of Microsatellite Polymorhism, SNP, and Mutation Cloning Analyses

	Case D1			Case D3			Case D4			Case D8			Case D9			Case D10			Case M4		
**SNP**	**F**	**C**	**M**	**F**	**C**	**M**	**F**	**C**	**M**	**F**	**C**	**M**	**F**	**C**	**M**	**F**	**C**	**M**	**F**	**C**	**M**
rs12596341	C/C	C	A/A	ND	ND	ND	ND	ND	ND	ND	ND	ND	ND	ND	ND	ND	ND	ND	ND	ND	ND
rs10660430	-/ATG	-	ATG/ ATG	ND	ND	ND	ND	ND	ND	ND	ND	ND	ND	ND	ND	ND	ND	ND	ND	ND	ND
rs11398689	-/G	-	G/G	ND	ND	ND	ND	ND	ND	ND	ND	ND	ND	ND	ND	ND	ND	ND	ND	ND	ND
rs9941308	ND	ND	ND	ND	ND	ND	ND	ND	ND	ND	ND	ND	NI	NI	NI	C/C	C	G/G	ND	ND	ND
rs58016760	ND	ND	ND	ND	ND	ND	ND	ND	ND	ND	ND	ND	----/----	----	GTGT/ GTGT	NI	NI	NI	ND	ND	ND
rs1364225	NI	NI	NI	NI	NI	NI	C/C	C	G/G	C/C	C	G/G	ND	ND	ND	ND	ND	ND	ND	ND	ND
rs1424019	NI	NI	NI	NI	NI	NI	A/A	A	G/G	A/A	A	G/G	ND	ND	ND	ND	ND	ND	ND	ND	ND
rs1364224	NI	NI	NI	NI	NI	NI	NI	NI	NI	A/A	A	G/G	ND	ND	ND	ND	ND	ND	ND	ND	ND
rs1424016	NI	NI	NI	NI	NI	NI	NI	NI	NI	A/A	A	C/C	ND	ND	ND	ND	ND	ND	ND	ND	ND
rs58557724	NI	NI	NI	NI	NI	NI	T/T	T	C/C	T/T	T	C/C	ND	ND	ND	ND	ND	ND	ND	ND	ND
rs1064259	NI	NI	NI	NI	NI	NI	A/A	A	G/G	NI	NI	NI	ND	ND	ND	ND	ND	ND	ND	ND	ND
rs2078304	ND	ND	ND	ND	ND	ND	ND	ND	ND	ND	ND	ND	ND	ND	ND	ND	ND	ND	C/C	C/G	G/G
Association of mutation c.1063T>C with rs2078304	ND	ND	ND	ND	ND	ND	ND	ND	ND	ND	ND	ND	ND	ND	ND	ND	ND	ND	T/T	C with C T with G	T/T
**Microsatellite marker**				**F**	**C**	**M**															
Chr:84,723,490 -84,724,542				1,1	1	2,3															
**Origin**	**Maternal**			**Maternal**			**Maternal**			**Maternal**			**Maternal**			**Maternal**			**Paternal**		

ND, not determined; NI, not informative; F, Father; M, Mother; C, Child

Table S2. Associated Anomalies in ACD/MPV Patients without a Mutation in *FOXF1*

Case	Associated Anomalies
1.4	Congenital heart disease, severe coarctation of aorta, ventricular septal defect, mitral valve and tricuspid valve malformation, malformed atrial septum, patent ductus arteriosus, left ventricular hypoplasia
3.4	GI malrotation, pneumothorax
5.3	Bilateral periventricular leukomalacia, hydronephrosis of left kidney
7.3	Right lung with two lobes, bilateral pyelocaliceal dilatation with thinned renal cortices
10.3	Voluminous heavy lungs, both right and left lungs are bilobed
13.5	Hepatosplenomegaly
15.3	Butterfly vertebra, imperforate anus with mildly distended large bowel, two lobes on right and one on left lung, pulmonary, renal, adrenal, and splenic congestion
20.8	Cardiomegaly, right ventricular hypertrophy, autopsy limited to heart and lungs
21.6	Patient D8
28.7	Patient D10
30.4	Post mortem lung biopsy, no autopsy, very small placenta
38.3	Autopsy limited to lungs, fusion of right upper and middle lobes and left upper and lower lobes, bilateral pneumothoraces, acute bronchopneumonia of lower left lobe
43.4	Membranous pyloric atresia and bowel malrotation, small bowel adhesions, absence of gallbladder and quadrate lobe of liver, hepatosplenomegaly
47.4	Patient D9
